# Study protocol of a pilot study on sirolimus-coated balloon angioplasty in salvaging clotted arteriovenous graft

**DOI:** 10.1186/s42155-020-00123-4

**Published:** 2020-07-05

**Authors:** Ru Yu Tan, Chee Wooi Tan, Suh Chien Pang, Marjorie Wai Yin Foo, Tjun Yip Tang, Apoorva Gogna, Tze Tec Chong, Chieh Suai Tan

**Affiliations:** 1 Department of Renal Medicine, Singapore General Hospital, Academia, Level 3, 20 College Road, Singapore, 169856 Singapore; 2 Department of Vascular Surgery, Singapore General Hospital, Singapore, Singapore; 3 Department of Vascular and Interventional Radiology, Singapore General Hospital, Singapore, Singapore

**Keywords:** Drug-coated balloon, Sirolimus, Clotted, Arteriovenous graft, Haemodialysis

## Abstract

**Background:**

In arteriovenous graft (AVG) for haemodialysis, the primary cause of failure is venous stenosis of the graft-vein junction from neointimal hyperplasia (NIH), resulting in thrombosis. While interventions to salvage clotted AVG are known to have high clinical success rates, long-term patency rates have been suboptimal. Drug-coated balloon (DCB) has been used to treat stenosed arteriovenous access in recent years with encouraging results but data on its effect in clotted AVG is unavailable.

**Methods:**

This is an investigator-initiated, single-center, single-arm prospective pilot study to determine the safety and outcome of the sirolimus-coated balloon (SCB) in the salvage of thrombosed AVG. Twenty patients who undergo successful percutaneous thrombectomy will receive treatment with SCB at the graft vein junction. The patients will be followed-up for 6-months. The primary endpoint is the patency rates at 3-month while the secondary endpoints are the patency rates and the number of interventions needed to maintain patency at 6-month.

**Discussion:**

Unremitting efforts have been made to prolong the patency of AV accesses over the years. DCB angioplasty combines mechanical and biological treatment for vascular stenosis. Sirolimus, being a cystostatic anti-proliferative agent, has been successfully used in coronary artery interventions. As the primary pathology of vascular stenosis in the dialysis circuit is neointimal hyperplasia, the use of sirolimus in balloon angioplasty may be effective. With this prospective study, we evaluate the efficacy and safety of SCB in patients with clotted AVG.

**Trial registration:**

ClinicalTrials.gov Identifier: NCT03666208 on 11 September 2018.

## Background

In arteriovenous graft (AVG) for haemodialysis, the primary cause of failure is venous stenosis of the graft-vein junction from neointimal hyperplasia (NIH), resulting in thrombosis (Roy-Chaudhury et al. 2006). While interventions to salvage clotted AVG are known to have high clinical success rates, long-term patency rates have been suboptimal. The reported average cumulative patency rates of AVG following successful thrombolysis or mechanical thrombectomy at 3- and 6-month were 49% and 38%, respectively (Dariushnia et al. 2016). Based on this, the Society of Interventional Radiology recommended a patency threshold of 44% at 3-months and 31% at 6-month following successful thrombectomy (Dariushnia et al. 2016).

The process of neointimal hyperplasia is continuous in AVG, contributed by hemodynamic changes following anastomotic grafting, presence of the AVG itself as a foreign body provoking an inflammatory response, injury from repeated cannulations and endothelial dysfunction in the presence of uraemia (Roy-Chaudhury et al. 2006). In addition, mechanical injury during balloon angioplasty and thrombectomy may also accelerate neointimal hyperplasia. Repeated interventions are therefore often required to maintain the circuit patency of AVG.

Drug-coated balloon (DCB), a balloon catheter that delivers an anti-proliferative agent to target lesion during percutaneous angioplasty has been used to treat failing arteriovenous (AV) access in recent years with encouraging results. Paclitaxel-coated balloon (PCB) has been demonstrated in several recent randomized control trials (RCT) to be superior to plain balloon in the treatment of AV access stenosis (Irani et al. 2018; Swinnen et al. 2019; Kitrou et al. 2015; Trerotola et al. 2018). However, these trials generally have excluded patients with AVG and those with thrombosis. Whether DCB has a similar patency effect on clotted AVG is therefore unknown, necessitate the search for an answer. Sirolimus is another class of anti-proliferative agent that has low toxicity able to inhibit neointimal hyperplasia and has been successfully used in coronary artery interventions (Verheye et al. 2017). It is indicated for in-stent stenosis and small vessel disease has not been used previously in dialysis access interventions (Verheye et al. 2017; Jim et al. 2016). This study is therefore conducted to investigate the effect of sirolimus coated balloon in dialysis access interventions.

## Materials and methods

### Hypothesis, aims and design

We hypothesize that the application of SCB at the graft vein junction following successful endovascular thrombectomy minimized neointimal hyperplasia and improve AVG patency and aim to examine the efficacy and safety of SCB angioplasty of the graft vein junction following successful thrombectomy of clotted AVG.

This single-center, single-arm prospective pilot study is an investigator-initiated study that is conducted in accordance with the ethical principles that have their origin in the Declaration of Helsinki and is approved by the center’s Centralized Institutional Review Board (CIRB number: 2018/2233). Informed consent will be obtained from all subjects recruited.

### Study population

Patients with end-stage renal disease (ESRD) presenting with a thrombosed AVG since October 2018 are screened for eligibility. Eligible patients are offered participation. The potential benefits and risks of SCB are explained before informed consent is obtained from participants. A total of 20 patients will be recruited and followed up for 6 months after the intervention.

#### Inclusion criteria

- Age 21–85 years
- Thrombosed AVG in the arm
- Successful thrombolysis of the thrombosed AVG, defined as the re-establishment of flow on Digital Subtraction Angiography (DSA) and restoration of thrill in the AVG on clinical examination

#### Exclusion criteria

- Patient unable to provide informed consent
- Previous bare-metal stent or stent-graft placement within the dialysis access
- Presence of central vein stenosis
- Sepsis or active infection
- Recent intracranial bleed or gastrointestinal bleed within the past 12 months
- Allergy to iodinated contrast media, anti-platelet drugs, heparin or paclitaxel
- Pregnancy
- Life expectancy < 12 months based on physician’s estimate

Patient who was receiving anticoagulation was initially excluded from the study. This criterion was subsequently changed to increase the potential pool of patients by allowing patients receiving anticoagulation to be enrolled in the study.

### Investigational device

The sirolimus-coated balloon (EXTREME TOUCH NEO, Concept Medical Research Private Limited, India) under investigation is coated with Sirolimus homogeneously through spray coating. The total dose of Sirolimus corresponds to 1.25 μg/mm^2^ of the surface of the balloon. The sizes of balloons available for this study are of 7 and 8 mm in diameter with a balloon length of 8 cm and shaft length of 80 cm over a 0.035″ wire.

### Study procedure

The procedure is performed according to our center protocol as previously described (Tan et al. 2019a, b) at the Interventional Nephrology Suite of a tertiary hospital in Singapore, which performs over 1000 access salvage procedures a year. After restoration of flow and adequate pre-dilatation with plain balloon, angiogram will be performed to ensure satisfactory treatment and absence of vessel recoil (defined as < 30% residual stenosis compared to the healthy segment of the AVG), SCB is then applied at the culprit lesion after appropriate sizing using the diameter of the graft (6 mm) as reference. To ensure adequate contact, the SCB balloon size is chosen in a 1:1 ratio or oversized by 1 mm the diameter compared to the reference vessel. An inflation device with a pressure gauge is used to inflate the SCB to stated burst pressure for 2 min. The size of the balloon used, inflation pressure, the transit time of the balloon, number of inflations, procedure complications (if any), and residual stenosis are recorded. Recruited patients with unsuccessful thrombectomy or unsatisfactory treatment of the graft vein junction (> 30% residual stenosis) will be considered screen failure and excluded in the study.

Post-procedure, all patients receive daily oral doses of aspirin (100 mg) and clopidogrel (75 mg) for 1 month, followed by treatment with aspirin alone for 6 months. For patients who are already on aspirin before the procedure, they will continue aspirin after the trial. For patients who are already on clopidogrel before the trial, they will continue clopidogrel after 1 month of the combination therapy. For patients who are on anti-coagulation, single anti-platelet therapy will be used for 6 months.

### Follow-up

#### Immediate post-intervention

All participants will be followed-up after successful intervention to assess the clinical patency defined as at least one successful haemodialysis with the prescribed blood flow and immediate complications.

#### One-month follow-up

All participants will receive a call from study investigator to assess for localised infection/hematoma/bleeding of the AVG, compliance to antiplatelet therapy and identify any adverse effects from antiplatelet therapy and SCB angioplasty.

#### Three and six-month post-intervention clinic assessment

Follow-up review and assessment of AVG are performed at 3- and 6-month after the procedure. Problems with haemodialysis including difficulties with cannulation, prolong bleeding after needle removal, haematoma due to miscannulation, high venous pressure and inadequate dialysis clearance will be recorded. Ultrasound examination of the entire AVG circuit will be performed by one of the interventional nephrologists who is also the study member for any abnormalities. The minimum diameter of the graft vein junction and diameter of the adjacent healthy segment of the AVG are measured in B-mode and colour doppler mode to estimate the degree of stenosis. Three separate measurement in each mode will be obtained and the mean of these measurement will be taken as the final degree of stenosis. A significant stenosis is defined as stenosis of > 50%. At the same time, access flow is also determined from the mean of three flow measurement at the feeding brachial artery.

### Endpoints and definitions

#### Primary endpoint

1. The primary circuit patency rates of the AVG at 3-months following endovascular thrombectomy and SCB angioplasty

#### Secondary endpoints

1. The primary circuit patency rates of the AVG at 6-months following endovascular thrombectomy and SCB angioplasty
2. The assisted-primary and secondary circuit patency rates of the AVG at 3- and 6-months following endovascular thrombectomy and SCB angioplasty
3. The number of interventions needed to maintain patency in 6-months

#### Primary safety endpoint

1. Freedom from local or systemic adverse reaction with SCB angioplasty within the first month

The patency outcomes are classified according to the recommendations by the Society of Interventional Radiology (Gray et al. 2003). Postintervention primary patency is defined as interval following intervention until the next required intervention (angioplasty, thrombolysis or surgical revision) or time of measurement of patency. Postintervention assisted primary patency is defined as interval after intervention until subsequent access thrombosis or time of measurement of patency. Secondary patency was defined as interval after intervention until the access is abandoned or time of measurement of patency.

The study ends after post-intervention ultrasound assessment at 6-month. Patient will be considered to have completed the study if they require any intervention to the AVG or the AVG thrombosed before 6-month. Adverse reactions, deaths, hospital admissions, interventional procedure that occurred within 6-month following SCB angioplasty will be recorded and analysed for all patients. A repeat angiogram of the AVG will only be performed in patients who have not reached primary endpoints when thrombosis or problems with dialysis mentioned above occur upon assessment by patient’s primary physician.

### Sample size calculation and statistical analysis

The required sample size is estimated for the primary outcome variable using STATA (StataCorp.2019. Stata Statistical Software: Release 16. College Station, TX: StataCorp LLC). Based on the recommended 3-month primary patency of 44% following thrombectomy, we estimated that 3-month primary patency could improve to 75% following SCB treatment and estimated that the sample size needed is 20 subjects using an alpha risk of 0.05 and a power of 0.8.

The data analyses will be performed with STATA (StataCorp.2019. Stata Statistical Software: Release 16. College Station, TX: StataCorp LLC) and SPSS version 23 (IBM Corp, Armonk, NY). Patency rates of the AVG following SCB angioplasty will be estimated with Kaplan-Meier analysis.

## Discussions

Vascular access has always been referred to as the Achilles heel of haemodialysis. Percutaneous transluminal angioplasty (PTA) was introduced for the management of stenosis of vascular access in 1981 (Lawrence et al. 1981). This technique has since become the standard of care for the treatment of dysfunctional AV accesses but recurrent stenosis was soon discovered as the other Achilles heel for the maintenance of AV access patency (Beathard 1992, 2019).

Unremitting efforts have been made to prolong the patency of AV accesses over the years. High pressure and cutting balloons were found to be helpful to a certain extent but elastic recoil and accelerated NIH remain significant limitations (Aftab et al. 2014; Rasuli et al. 2015; Wakamoto et al. 2018). Stent-grafts have been shown in RCT to improve the patency rates but the cost has largely limited its use in many patients (Vesely et al. 2016). Moreover, the use of stent-graft may impede surgical revision of the AVG (Salman and Asif 2010). These proposed mechanical solutions however are not addressing the underlying biological changes contributing to NIH that leads to AV access dysfunction.

DCB technology is built to combine mechanical and biological treatment for vascular stenosis. The investigational DCB in this study uses Sirolimus, a cytostatic compound with a large therapeutic margin as its anti-proliferative agent compared to Paclitaxel, which has a narrow therapeutic margin and is cytotoxic (Wessely et al. 2006). Although there have been no long-term data on mortality associated with Paclitaxel use in AV access as this technique is still considered relatively new in dialysis access management, a meta-analysis by Kennedy et al. reported a higher 12-month mortality of 7.6% vs. 5.8% for PCB angioplasty compared to plain balloon angioplasty although it did not reach statistical significance (Kennedy et al. 2019). SCB therefore has the theoretical safety advantage against PCB. Although both Sirolimus and Paclitaxel were found to be effective in retarding endothelial regeneration, Sirolimus is equally distributed in the vessel layers in contrast to Paclitaxel, which accumulates in the adventitia and is believed to play an inferior role in preventing restenosis compared to Sirolimus (Wessely et al. 2006).

Clinically, systemic use of sirolimus in organ transplantation appears to impair wound healing. Prolong endothelial dysfunction has been found on coronary arteries treated with Sirolimus-eluting stent and aneurysms formation following Sirolimus-eluting stent placements have been reported in coronary arteries (Hofma et al. 2005; Zbinden et al. 2008). While endothelial dysfunction plays an important role in the formation of aneurysms in the arteries, the contributory role of Sirolimus cannot be ascertain. Hence, ultrasound was used as an assessment tool during the follow-up in this study so that any abnormalities in the AVG and draining veins particularly aneurysm or pseudoaneurysm formation can be detected and recorded.

Thrombosed AVG is chosen to investigate the effect of sirolimus in this pilot study because the patency rate is dismal post thrombectomy with plain balloon and we hope to find new therapeutic options that can potentially change clinical practice (Dariushnia et al. 2016). This study is limited by its small sample size. Being a single center study also limit its generalizability. In addition, assessment of recoil within the first few hours following SCB angioplasty was not performed and may result in failure to determine if the stenosis seen on ultrasound assessment at 3- or 6-month part of progression or recoil post-angioplasty. However, the results of this study will help in hypothesis generating and fine-tuning of protocol in a larger scale, multi-center randomized controlled trial to confirm the efficacy and safety of SCB in dialysis access in near future.

## Data Availability

The datasets generated and/or analyzed during the current study are not publicly available due to confidentiality of the data but are available from the corresponding author on reasonable request.

## Abbreviations

AVG: Arteriovenous graft
NIH: Neointimal hyperplasia
DCB: Drug-coated balloon
SCB: Sirolimus-coated balloon
AV: Arteriovenous
PCB: Paclitaxel-coated balloon
ESRD: End stage renal disease
DSA: Digital Subtraction Angiography
PTA: Percutaneous transluminal angioplasty

## References

[CR1] Aftab SA, Tay KH, Irani FG, Gong Lo RH, Gogna A, Haaland B (2014). Randomized clinical trial of cutting balloon angioplasty versus high-pressure balloon angioplasty in hemodialysis arteriovenous fistula stenoses resistant to conventional balloon angioplasty. J Vasc Interv Radiol.

[CR2] Beathard GA (1992). Percutaneous transvenous angioplasty in the treatment of vascular access stenosis. Kidney Int.

[CR3] Beathard GA (2019). Techniques for angioplasty of the arteriovenous hemodialysis access. UpToDate.

[CR4] Dariushnia SR, Walker TG, Silberzweig JE, Annamalai G, Krishnamurthy V, Mitchell JW (2016). Quality improvement guidelines for percutaneous image-guided management of the thrombosed or dysfunctional dialysis circuit. J Vasc Interv Radiol.

[CR5] Gray RJ, Sacks D, Martin LG, Trerotola SO, Society of Interventional Radiology Technology Assessment C (2003). Reporting standards for percutaneous interventions in dialysis access. J Vasc Interv Radiol.

[CR6] Hofma SH, van der Giessen WJ, van Dalen BM, Lemos PA, McFadden EP, Sianos G (2005). Indication of long-term endothelial dysfunction after sirolimus-eluting stent implantation. Eur Heart J.

[CR7] Irani FG, Teo TKB, Tay KH, Yin WH, Win HH, Gogna A (2018). Hemodialysis arteriovenous fistula and graft stenoses: randomized trial comparing drug-eluting balloon angioplasty with conventional angioplasty. Radiology.

[CR8] Jim MH, Fung RC, Yiu KH (2016). Angiographic result of sirolimus-eluting balloon in de novo small coronary artery lesion (ARSENAL). Int J Cardiol.

[CR9] Kennedy SA, Mafeld S, Baerlocher MO, Jaberi A, Rajan DK (2019). Drug-coated balloon angioplasty in hemodialysis circuits: a systematic review and meta-analysis. J Vasc Interv Radiol.

[CR10] Kitrou PM, Spiliopoulos S, Katsanos K, Papachristou E, Siablis D, Karnabatidis D (2015). Paclitaxel-coated versus plain balloon angioplasty for dysfunctional arteriovenous fistulae: one-year results of a prospective randomized controlled trial. J Vasc Interv Radiol.

[CR11] Lawrence PF, Miller FJ, Mineaud E (1981). Balloon catheter dilatation in patients with failing arteriovenous fistulas. Surgery.

[CR12] Rasuli P, Chennur VS, Connolly MJ, Hadziomerovic A, Pomerleau FE, Ryan SE (2015). Randomized trial comparing the primary patency following cutting versus high-pressure balloon angioplasty for treatment of de novo venous stenoses in hemodialysis arteriovenous fistulae. J Vasc Interv Radiol.

[CR13] Roy-Chaudhury P, Sukhatme VP, Cheung AK (2006). Hemodialysis vascular access dysfunction: a cellular and molecular viewpoint. J Am Soc Neph.

[CR14] Salman L, Asif A (2010). Stent graft for nephrologists: concerns and consensus. Clin J Am Soc Nephrol.

[CR15] Swinnen JJ, Hitos K, Kairaitis L, Gruenewald S, Larcos G, Farlow D (2019). Multicentre, randomised, blinded, control trial of drug-eluting balloon vs Sham in recurrent native dialysis fistula stenoses. J Vasc Access.

[CR16] Tan RY, Pang SC, Teh SP, Lee KG, Chong TT, Gogna A (2019). Comparison of alteplase and urokinase for pharmacomechanical thrombolysis of clotted hemodialysis access. J Vasc Access.

[CR17] Tan Ru Yu, Pang Suh Chien, Teh Swee Ping, Ng Chee Yong, Lee Kian Guan, Foo Marjorie Wai Yin, Gogna Apoorva, Chong Tze Tec, Tan Chieh Suai (2020). Outcomes of endovascular salvage of clotted arteriovenous access and predictors of patency after thrombectomy. Journal of Vascular Surgery.

[CR18] Trerotola SO, Lawson J, Roy-Chaudhury P, Saad TF (2018). Drug coated balloon angioplasty in failing AV fistulas: a randomized controlled trial. Clin J Am Soc Nephrol.

[CR19] Verheye S, Vrolix M, Kumsars I, Erglis A, Sondore D, Agostoni P (2017). The SABRE Trial (Sirolimus Angioplasty Balloon for Coronary In-Stent Restenosis): angiographic results and 1-year clinical outcomes. JACC Cardiovasc Inter.

[CR20] Vesely T, DaVanzo W, Behrend T, Dwyer A, Aruny J (2016). Balloon angioplasty versus Viabahn stent graft for treatment of failing or thrombosed prosthetic hemodialysis grafts. J Vasc Surg.

[CR21] Wakamoto K, Doi S, Nakashima A, Kawai T, Kyuden Y, Naito T (2018). Comparing the 12-month patency of low- versus high-pressure dilation in failing arteriovenous fistulae: a prospective multicenter trial (YOROI study). J Vasc Access.

[CR22] Wessely R, Schömig A, Kastrati A (2006). Sirolimus and paclitaxel on polymer-based drug-eluting stents: similar but different. J Am Coll Cardiol.

[CR23] Zbinden R, Eshtehardi P, Cook S (2008). Coronary aneurysm formation in a patient early after everolimus-eluting stent implantation. J Invasive Cardiol.

